# Diagnostic Scoring in Biliary Atresia*

**DOI:** 10.5152/tjg.2025.24469

**Published:** 2025-05-20

**Authors:** Şükrü Güngör, Fatma İlknur Varol, Ebubekir Altundaş, Emre Gök, Turan Yıldız, Sevgi Demiröz

**Affiliations:** 1 Department of Pediatric Gastroenterology, İnönü University School of Medicine, Malatya, Türkiye; 2 Department of Pediatric Surgery Clinic, İnönü University School of Medicine, Malatya, Türkiye; 3 Department of Pediatric Radiology, İnönü University School of Medicine, Malatya, Türkiye

**Keywords:** Biliary atresia, children, diagnosis, score

## Abstract

**Background/Aims:**

The aim of this study was to develop a diagnostic scoring model to predict the need for intraoperative cholangiography in patients with neonatal cholestasis suspected of having biliary atresia (BA) and to aid in the early diagnosis of BA.

**Materials and Methods:**

Data from 70 patients with neonatal cholestasis who underwent intraoperative cholangiography with a preliminary diagnosis of BA between 2019 and 2024 were retrospectively reviewed. Data from patients with and without BA were compared. Thescoring was based on 3 parameters: acholic stool observed clinically on inspection, findings suggestive of BA on ultrasound, and elevated gamma-glutamyl transferase (GGT) levels. The best GGT cut-off point for the diagnosis of BA was determined by receiver operating characteristic analysis. The diagnostic success of the scoring model for BA was statistically evaluated.

**Results:**

There were no significant differences in age and gender between BA and non-BA groups. Gamma-glutamyl transferase levels were elevated in all patients. Acholic stools were present in 98% of BA patients. Ultrasound findings suggestive of BA were present in 88.5% of patients with BA. The authors found the best GGT cut-off value for the diagnosis of BA to be ≥366 (73% sensitivity, 77.8% specificity). In the scoring model the authors developed, the presence of 2 parameters provided diagnostic success with high sensitivity (98%) and specificity (83.3%).

**Conclusion:**

The study provides a reliable and sensitive diagnostic criterion to determine the need for intraoperative cholangiography in infants with neonatal cholestasis. These data should be validated in larger prospective case series.

**Main Points**

- Early diagnosis of biliary atresia saves lives. The scoring enables early diagnosis.
- The study will help determine the need for intraoperative cholangiography in patients with neonatal cholestasis.
- The study will contribute to the success of surgery with early diagnosis in patients requiring portoenterostomy surgery.

## Introduction

Biliary atresia (BA), the most common cause of liver transplantation in children, is an idiopathic, fibroobliterative, and progressive disease of the extrahepatic biliary tree that occurs in the neonatal period. Biliary atresia accounts for one-fourth of neonatal cholestasis. The incidence is 0.5-2/10 000.^1,2^ In 10%-20% of cases, BA is associated with other congenital malformations such as situs inversus and asplenia. It has been reported that the prognosis of BA with these malformations may be worse.^1^^-^^5^ Babies with BA are usually born at term with a normal birth weight. They develop normally at first. Jaundice develops within the first 8 weeks. It is unlikely to develop later. Physical examination often reveals acholic stools, dark urine, and organomegaly.^5^^-^^8^ There is no single clinical or laboratory marker for the diagnosis of BA. Stool color cards have been used in clinical screening for acholic feces in the neonatal period. However, their sensitivity is low (sensitivity 76.5%, specificity 99%).^7^^-^^9^ Although the presence of the triangular zone sign on ultrasound is helpful in the diagnosis, it may not be seen in all patients with BA. Some studies^10^^,^^11^ emphasize that the triangular zone sign is a result of diffuse fibrosis and appears after the development of cirrhosis and is even a negative marker for surgical success. The sensitivity of hepatobiliary scintigraphy in the diagnosis and exclusion of BA is high (98.7%), but the specificity is low (37%-74%).^12^ The diagnostic accuracy of hepatobiliary scintigraphy for BA is 67%.^12^ It can give false positive results in liver disease with severe hepatocellular damage. Therefore, passage of radioactive material into the intestine excludes BA, whereas absence of passage does not confirm BA.^12^ Exposure of the patient to radioactive material, difficulty in obtaining hepatobiliary iminodiacetic acid (HIDA) and long waiting times for an appointment are other factors limiting its use.

The differential diagnosis of BA without intraoperative cholangiography or the identification of patients requiring intraoperative cholangiography has been the subject of research for many years. The development of cheap, reliable, and readily available diagnostic models is still ongoing.

Early diagnosis of BA will both prevent the rapid development of liver failure and save time for successful liver transplantation. Therefore, a simplified diagnostic scoring model was aimed to be developed based on clinical, biochemical, and ultrasonographic (US) findings in patients with and without BA diagnosed by intraoperative cholangiography.

## Materials and Methods

### Patient Selection

This study is a cross-sectional cohort study in which the data of 497 infants diagnosed with cholestasis between 2019 and 2024 were retrospectively examined. Of these, 70 patients who underwent intraoperative cholangiography with a pre-diagnosis of BA were included in the study. Of the patients in question, 52 (74.3%) exhibited BA, while 18 (25.7%) did not. Patients were divided into 2 groups.

Group 1: Patients in whom the diagnosis of BA was excluded by intraoperative cholangiography.

Group 2: Patients in whom BA was diagnosed by intraoperative cholangiography.

The study was approved by İnonu University Medical Faculty Hospital Non-Interventional Clinical Research Ethics Committee (Approval date: July 30, 2024, decision number: 2024/6155). Thestudy was complied with the Declaration of Helsinki. Since it was a retrospective study, informed consent forms were not obtained from the cases.

### Patient Data

#### Demographic Characteristics:

Age and gender.

### Clinical Findings

Acholic stools, splenomegaly, hepatomegaly, and presence of ascites.

### Ultrasound Findings Suggestive of Biliary Atresia^11^^,^^13^^,^^14^

Triangular zone, echogenicity >4 mm anterior to right portal vein.

Gallbladder atresia, abnormal or dysmorphic gallbladder.

Failure to visualize the common bile duct.

The gallbladder is contracted.

Failure to visualize the gallbladder.

### Biliary Atresia Findings on Intraoperative Cholangiography^15^

Passing of contrast material administered into the gallbladder into the bowel and intrahepatic bile ducts rules out BA.

In the presence of a fibrotic gallbladder and/or an atretic extrahepatic biliary tree, BA is diagnosed without cholangiography, and a Kasai portoenterostomy is performed. 

Failure of intraoperative cholangiography to visualize the biliary tree leads to the diagnosis of BA.

### Scoring

The presence of findings suggestive of BA, as listed in the Table below, was scored as 1 and their absence as 0.

Receiver operating characteristic (ROC) curve analysis was performed on the scores obtained with the BA criteria. The best cut-off point for predicting BA was determined. Biliary atresia was diagnosed in patients with 2 of the 3 criteria (>1.5 points) with a sensitivity of 98% and a specificity of 83.3%.

### Inclusion Criteria

Pediatric patients aged 1 month-6 months undergoing intraoperative cholangiography with a pre-diagnosis of BA were included in the study.

### Exclusion Criteria

Patients for whom intraoperative cholangiography data were not available

Patients in whom BA was suspected based on clinical, laboratory, and pathological data but in whom intraoperative cholangiography was not performed,

Patients who underwent intraoperative cholangiography but for whom preoperative clinical and laboratory data were not available were not included in the study.

### Statistical Analysis

Analyses were performed using the Statistical Package for the Social Sciences for Windows 22 (IBM SPSS Corp.; Armonk, NY, USA). Statistical variables were expressed as number, mean, standard deviation, and percentage. The Kolmogorov–Smirnov test was used for normality analysis of numerical variables. Student’s *t*-test or one-way analysis of variance (ANOVA) was used to analyze numerical data that showed a normal distribution. Kruskal–Wallis or Mann–Whitney *U* test was used for non-normally distributed parameters. Independent Student *t*-test was used to determine whether there was a significant difference between the arithmetic means of the dependent variables between the group with and without BA. Receiver operating characteristic curve analysis was performed to determine the diagnostic cut-off points of the gamma-glutamyl transferase (GGT) and the BA score data that could predict atresia. Risk analysis for BA was performed using logistic regression test for the parameters for which cut-off points were determined. *P* < .05 was considered significant.

## Results

The records of 497 patients with cholestasis were retrospectively reviewed. Of these, 70 patients who underwent intraoperative cholangiography at thecenter were included in the study. Biliary atresia was excluded by cholangiography in 18 patients (25.7%). Familial intrahepatic cholestasis was diagnosed in 6 patients (33.3%) (3 patients had [Progressive Familial Intrahepatic Cholestasis] PFIC 2, 2 patients had PFIC 3, and 1 patient had PFIC 1), Caroli disease in 3 (16.6%), alpha-1 antitrypsin deficiency in 2 (11%), Alagille syndrome in 2 (11%), neonatal hepatitis in 2 (11%), Cytomegalovirus (CMV) infection in 1 (5.5%), and idiopathic in 2 (11%). Biliary atresia was diagnosed in 52 patients (74.3%) and Kasai surgery was performed. All patients underwent liver biopsy during intraoperative cholangiography.

When the demographic and clinical findings of the patients with and without BA diagnosis were evaluated, there was no significant difference between the 2 groups in terms of age and gender. The mean age of the patients included in the study at the time of diagnosis was 59 ± 21.57 days. There was no significant difference in age and gender between the groups (Tables 1 and 2).

Acholic feces was present in 98% of patients with BA and in half of the group without BA. A US finding suggestive of BA was present in 88.5% of patients with BA. This rate was significantly higher than in the group without BA (*P* < .001). The most common US finding was gallbladder contraction (63.5%).

In terms of histopathological findings, portal fibrosis was more common in the BA group (80.8%). The rate of other pathological findings was not different between the groups. Splenomegaly was significantly higher in the BA group (*P* : .001) (Table 2).

When the patients’ laboratory data were compared between groups, GGT, total protein, platelet distribution width and globulin values were significantly higher in the BA group. There were no significant differences between the groups in other laboratory parameters (Table 2). The BA score was significantly higher in the BA group (*P* < .001).

The diagnostic cut-off point for BA in GGT and BA score was evaluated by ROC curve analysis. It was found that GGT ≥366 predicted BA with 73% sensitivity, 77.8% specificity, 50% negative predictive value, and 90.5% positive predictive value. Biliary score ≥1.5 predicted BA with 98% sensitivity, 83.3% specificity, 93.7% negative predictive value, and 94.4% positive predictive value (Table 3).

When the risk factors associated with BA were evaluated by logistic regression analysis, it was shown that the presence of acholic feces 51-fold, GGT ≥366 8.6-fold, presence of US findings suggestive of BA 20-fold, and BA score ≥1.5 increased the risk of BA 255-fold (Table 4).

## Discussion

The success of surgical repair of BA with portoenterostomy depends on early diagnosis. In order to differentiate BA from other cholestatic causes, it is necessary to observe the openings of the bile ducts and their passage into the duodenum.^15^ In the study by Sira et al,^15^ the rate of patients in whom BA was not diagnosed by intraoperative cholangiography was reported to be approximately 7%. In thestudy, this rate was 25%.

In the study by Sira et al,^15^ acholic stool and GGT values were not different between groups. In the study, GGT levels were normal for age in 5 patients. Two of these patients were diagnosed with BA and 3 were not. All other patients had high GGT levels for their age. Gamma-glutamyl transferase was significantly higher in patients with BA. These differences may be due to differences in clinical factors affecting GGT, such as age, sex, infections, and medication.

Acholic stools were absent in 10 (14.3%) of the patients included in the study. As these patients had US findings suggestive of BA and elevated GGT, intraoperative cholangiography was performed. Biliary atresia was diagnosed in only 1 of these patients. In thestudy, the rate of acholic stools was significantly higher in the BA group. In the study by Sira et al,^15^ approximately 10% of patients diagnosed with BA did not have acholic stools. However, they reported that all patients without BA had acholic stools. While no patient had ascites in thestudy, 16 patients with BA had ascites in the study by Sira et al.^15^ In the study, splenomegaly was observed at a significantly higher rate in patients with BA. Hepatomegaly did not differ between the 2 groups. In the study by Sira et al,^15^ hepatomegaly was significantly higher, whereas splenomegaly was not different. These differences may be due to clinical differences in the non-BA cholestasis patients included in the study.

In this study, US findings suggestive of BA were significantly higher in the group of patients with BA. This result supports the study of Sira et al.^15^ In this study, globulin levels were also compared among the biochemical data. It was found that it was significantly higher in the BA group. This suggests that immune-mediated events may play a role in the pathogenesis of BA.

Recently, there are studies in the literature on biomarkers with high sensitivity (95%-97%) and specificity (95%-98%) for BA (interleukin-33 and matrix metalloproteinase-7).^16^^-^^21^ The reason why the sensitivity and specificity in these studies are higher than in our study is that the healthy group and the cholestatic group were compared. In thisstudy, unlike the previous study, patient groups who were considered to have BA and therefore underwent intraoperative cholangiography were compared. The patient population that is often confused with BA in terms of clinical and laboratory findings in the differential diagnosis is reflected in this study.

It has been suggested that different types of serum bile acids may be present in cases where enterohepatic circulation is blocked.^22^^,^^23^ and attempts have been made to create a diagnostic score using multiple biomarkers, including clinical features and bile acid levels, to define BA. In this prospective study by Zhao et al,^24^ a 3-variable scoring system (bile acid levels, GGT, and acholic stool) was developed to define BA in cholestatic infants.^24^ The AUC of this combination was 0.89. It has been reported to be useful in the differential diagnosis of BA in cholestatic infants. However, the inability to determine bile acid levels in most centers, the high cost and the late analysis of results limit the applicability of this scoring model. A 3-variable, inexpensive, easy-to-use, and accessible diagnostic scoring system with clinical (acholic stool), laboratory (GGT), and Ultrasonography (USG) images is described in this study. The AUC of thescoring system was found to be 0.93.

Muthukanagarajan et al^25^ reported that the presence of histopathological features such as ductal plate malformation, high-grade fibrosis, bile duct proliferation, and cholestasis in extrahepatic BA was strongly associated with poor prognosis. However, they could not detect any histopathological findings that could diagnostically distinguish BA from other neonatal cholestases. Liver biopsy is recommended in the diagnostic process of BA in cases where supporting histopathological findings need to be demonstrated and differentiation from intrahepatic biliary tract pathologies that do not require surgical intervention is required.^25^^-^^27^ The literature is supported by the study, and it wasfound that portal fibrosis was significantly higher in the group of patients with BA. There was no significant difference in other histopathological findings between the 2 groups.

Liu et al^28^ developed a non-invasive diagnostic scoring system including biochemical markers and hepatobiliary scintigraphy findings. They reported that the scoring system they developed could differentiate BA with 93.8% sensitivity and 96.0% specificity. However, the lack of HIDA required for hepatobiliary scintigraphy in the country, the exposure of patients to radioactive substances and the long appointment times for scintigraphy limit its preferability. For this reason, scintigraphic findings were not included in the diagnostic scoring in the study. The scoring system that wasdeveloped is a radiation-free, easily available and inexpensive method. A sensitivity of 98%, specificity of 83.3%, positive predictive value of 94.4%, and negative predictive value of 94% were found.

Liu et al^29^ created a diagnostic nomogram based on acholic stool, gallbladder length, gallbladder emptying index, shear wave elastography value and GGT level. They reported that this nomogram had high diagnostic sensitivity and specificity for BA in 2-3 month old infants. The study is different in that elastography data is not included. However, it is a simpler and cheaper diagnostic model and has high sensitivity and specificity values.

The fact that this study is a retrospective study may have caused bias due to the lack of materials required for scoring or difficulty in accessing data, and thus the limited number of cases. This is the most important limiting aspect of the study.

As a result, the study provides diagnostic criteria with high reliability and sensitivity to help clinicians select patients who require intraoperative cholangiography and/or Kasai surgery in infants <3 months with cholestasis. These findings need to be confirmed by prospective studies with larger case series.

## Figures and Tables

**Table:** 

Findings	Available	Nonavailable	Score
Acholic feces	1	0	
Hepatobiliary US finding suggestive of BA	1	0	
GGT elevation (>366 IU/L)	1	0	
**Total score**			

**Table 1.** Evaluating the Differences in Patients’ Demographic and Clinical Findings Between Groups

		Cholestasis Without Biliary Atresia (18) n (%)	Biliary Atresia (52) n (%)	*P*
Gender	Female (36)	8 (44.4)	28 (53.8)	.492
	Male (34)	10 (55.6)	24 (46.2)	
Acholic stool		9 (50)	51 (98.1)	<.001
BA finding on US (70)		5 (27.8)	46 (88.5)	<.001
Abdominal US	Normal	13 (72.2)	6 (11.5)	<.001
	Gallbladder contracted	5 (27.8)	33 (63.5)	
	Gallbladder could not be visualized	0 (0)	13 (25)	
Histopathological findings	Portal fibrosis (70)	10 (55.6)	42 (80.8)	.035
	Bile duct proliferation (70)	12 (66.7)	31 (59.6)	.596
	Bile plug (70)	9 (50)	30 (57.7)	.571
	Cholestasis (70)	10 (55.6)	38 (73.1)	.168
	Hepatocyte degeneration (67)	4 (23.5)	13 (19.4)	.840
	Inflammation in the portal area (66)	4 (23.5)	24 (36.3)	.513
	Extramedullary hematopoiesis (66)	2 (12.5)	5 (10)	.777
	Ductal plate malformation (66)	4 (25)	13 (26)	.937
Choledochal cyst		0 (0)	4 (7.8)	.221
Hepatosteatosis		1 (5.6)	3 (5.9)	.959
Ex		1 (5.6)	13 (25.5)	.071
Situs inversus		0 (0)	1 (1.5)	.541
Hepatomegaly		15 (83.3)	49 (94.2)	.155
Splenomegaly		12 (66.7)	50 (96.2)	.001

BA, Biliary atresia; US, ultrasonography.

**Table 2.** A Comparison of the Laboratory Findings Between the 2 Groups

	Cholestasis Without Biliary Atresia (18) Mean ± SD	Biliary Atresia (52) Mean ± SD	*P*
Age (year)	62.5 ± 20.04	57.76 ± 22.31	.430
AST (U/L)	326.61 ± 210.46	229.68 ± 135.87	.082
ALT (U/L)	182.83 ± 98.76	147.07 ± 119.77	.260
GGT (IU/L)	355.56 ± 412.15	731.21 ± 571.42	.013
ALP (U/L)	716.05 ± 340.96	724.41 ± 309.95	.924
Total bilirubin (mg/dL)	10.60 ± 4.04	10.95 ± 4.87	.787
Direct bilirubin (mg/dL)	6.82 ± 2.96	7.64 ± 3.83	.414
Total protein (g/dL)	5.34 ± 0.67	5.86 ± 0.72	.008
Albumin (g/dL)	3.14 ± 0.39	3.22 ± 0.54	.566
Hb (g/dL)	10.61 ± 1.21	12.70 ± 15.42	.570
WBC (10^3^/µL)	13.48 ± 5.30	13.78 ± 4.91	.830
PLT (10^3^/µL)	427.56 ± 145.91	435.72 ± 213.25	.881
MPV (fL)	10.36 ± 0.87	10.13 ± 1.69	.479
PDW (fL)	12.44 ± 2.74	14.26 ± 3.11	.032
GI (10^3^/µL)	0.077 ± 0.12	0.114 ± 0.322	.642
G (%)	0.497 ± 0.880	0.677 ± 1.960	.712
PT (%)	83.57 ± 35.33	76.12 ± 37.86	.472
PTT (sn)	14.89 ± 7.77	12.13 ± 5.28	.105
INR	1.13 ± 0.30	0.99 ± 0.41	.201
Globulin (g/dL)	2.19 ± 0.69	2.64 ± 0.78	.032
Biliary atresia score	1 ± 0.76	2.57 ± 0.53	<.001

İndependent student *t*-test.

ALP, alkaline phosphatase; ALT, alanine aminotransferase; AST, aspartate transferase; GGT, gamma-glutamyl transferase; GI, granulocyte index; G%, percentage of granulocytes; Hb, hemoglobin; INR, international normalized ratio; MPV, mean platelet volume; PDW, platelet distribution width; PLT, platelet; PT, prothrombin time; PTT, partial thromboplastin time; WBC, white blood cell.

**Table 3.** Determining the Best Cut-Off Points for Biliary Atresia

	Best Cut-Off Point for Biliary Atresia	Area Under the Curve	Sensitivity	Specificity	95% CI	Negative PV	Positive PV	*P*
GGT (IU/L)	≥366	0.749	0.730	0.778	0.632-0.845	50	90.5	<.001
Biliary score	≥1.5	0.931	0.98	0.833	0.846-1.000	93.7	94.4	<.001

ROC curve analysis.

GGT, gamma-glutamyl transferase; PV, predictive value.

**Table 4.** Examination of the Risk Factors Associated with Biliary Atresia

	OD	95% CI	*P*
Biliary atresia finding on ultrasonography	20	5.235-75.897	<.001
Acholic stool	51	5.741-453.030	<.001
GGT ≥366 (IU/L)	8.6	2.442-30.522	<.001
Biliary score	255	24.681-2634.655	<.001

Logistic regression analysis.

GGT, gamma-glutamyl transferase; OD, odds ratio.

## Data Availability

The data that support the findings of this study are available on request from the corresponding author.
